# Assessment of the Brazilian Portuguese Version Selective Control Assessment of the Lower Extremity (SCALE) After Translation and Cross-Cultural Adaptation

**DOI:** 10.3390/children13010115

**Published:** 2026-01-13

**Authors:** Douglas Manuel Carrapeiro Prina, Elizabeth de Alvarenga Borges da Fonseca, Pothyra Campos Pascoal, Francesco Camara Blumetti, Monica Paschoal Nogueira

**Affiliations:** 1 Instituto de Assistência Médica Ao Servidor Público Estadual (IAMSPE), São Paulo 04039-000, Brazil; fonseca.elizabeth@hotmail.com (E.d.A.B.d.F.); pothyracampos@gmail.com (P.C.P.); 2 Hospital Israelita Albert Einstein, São Paulo 05652-900, Brazil; francesco.blumetti@einstein.br; 3 Núcleo de Apoio à Pesquisa Ortopédica Avançada (NAPOA), São Paulo 04078-010, Brazil

**Keywords:** translational medical research, cerebral palsy, motor skills

## Abstract

**Highlights:**

**What are the main findings?**

• The Portuguese version of the Selective Control Assessment of the Lower Extremity (SCALE-BR) was successfully translated, cross-culturally adapted, and validated, proving to be a reproducible and reliable tool.
• SCALE-BR showed excellent internal consistency, intra- and interobserver reliability, and a strong, significant inverse correlation with the Gross Motor Function Classification System (GMFCS).

**What are the implications of the main findings?**

• SCALE-BR provides a standardized and clinically useful tool for the quantitative assessment of selective motor control in Brazilian patients with spastic cerebral palsy, supporting clinical decision-making and progress monitoring.
• This validation confirms selective motor control as a key factor in functional severity and mobility in cerebral palsy patients, which can refine therapeutic intervention selection and enhance understanding of gait.

**Abstract:**

**Background/Objectives**: This study aims to translate and validate the Selective Control Assessment of the Lower Extremity (SCALE) tool from English into Portuguese. **Methods**: SCALE was translated into Portuguese independently by two native Portuguese translators and synthesized into a single manuscript: SCALE-BR. Using this test in patients with spastic cerebral palsy, the internal consistency (Cronbach’s α), reliability by intra-class correlation (ICC), and validity compared with the Gross Motor Function Classification System (GMFCS) scores were evaluated. **Results**: 30 patients diagnosed with spastic cerebral palsy were assessed, with a predominance of males (66.7%), a mean age of 12.9 ± 7.9 (4–38 years) and a majority of diparetic patients (73.3%) and GMFCS I (53.3%). Spearman’s correlation coefficient, R^2^ = −0.84, *p* < 0.001, revealed an inverse relationship between the SCALE instrument and the GMFCS, corroborating the findings in the literature. There was an excellent intra- and interobserver agreement (ICC > 0.75). **Conclusions**: The Portuguese version of the questionnaire was effective, proving to be reproducible and reliable among different evaluators and patients, with an inverse correlation with the GMFCS as expected in the literature.

## 1. Introduction

Cerebral palsy (CP) is a group of permanent disorders of movement and posture, causing activity limitation, due to non-progressive disturbances in the developing fetal or infant brain, often accompanied by sensory, cognitive, communication, behavioral, epileptic, and musculoskeletal issues [1]. CP is the most prevalent mobility disorder in children, nearly 2.11 per 1000 live births, and has remained constant in recent years [1,2,3]. In Brazil, CP is one of the leading causes of physical disability in childhood, affecting a large population that requires comprehensive and effective rehabilitation interventions. The spastic form is the most common and is caused by damage to the corticospinal tract through which descending neural pathways (excitatory, and inhibitory) pass. As a result, there are four basic neuromuscular deficiencies: shortening of the musculotendinous unit, muscle weakness, spasticity, and impairment of selective motor control [4].

Recently, among the disorders found, selective motor control (SMC) has begun to be more studied. This is defined as isolated muscle activation in a selected pattern, in response to postural or voluntary movement demands, generated by instruction, imitation or preparation [5], being controlled both in relation to directionality and force generation [6]. It is an essential element for normal movement that allows for agility and independent joint control [7]. Current evidence suggests that SMC capacity is an important factor for functional movement tasks [8,9,10] and can be predictive of improvement in planning and post-intervention assessments [6,8,11]. The functional implications of compromised SMC are profound and directly affect the quality of life of patients and their families. Deficits in SMC transcend mere difficulty in performing isolated movements, manifesting themselves in specific gait abnormalities, such as the persistence of compensatory synergistic movement patterns, reduced speed and efficiency of locomotion, and poor postural control. Such limitations result in significant restrictions in participation in daily activities, challenges in independent mobility, and may contribute to the development of orthopedic deformities and increased risk of falls, compromising the individual’s social development and autonomy.

The importance of studying SMC lies in the history of different results after the same interventions in patients classified equally on the others motor scores, such as the GMFCS. The SMC is interpreted as a modifying factor for the outcome of procedures and therapies performed on these patients.

Initially, it was assessed indirectly and rudimentarily without standardized scores, mainly to predict the prognosis of functionality after selective dorsal rhizotomy [8,12]. In 2009, the Selective Control Assessment Lower Extremity (SCALE) test was developed [13]. This tool was created to examine the clinical and observational grading of SMC in patients with spastic CP.

The purpose of this study was to translate and culturally adapt the SCALE from English into Brazilian Portuguese, following international guidelines [14,15,16]. This research is not only a methodological advance but also a pressing clinical need, enabling accurate assessment of selective motor control (SMC) and supporting more targeted, personalized interventions to optimize functional outcomes and improve the quality of life of children and adolescents with cerebral palsy (CP) in Brazil. In addition, this study examines the reliability and validity of the version in Portuguese (SCALE-BR) for patients with spastic cerebral palsy.

## 2. Materials and Methods

### 2.1. Instrument

The SCALE was designed by Fowler et al. [13] to evaluate the selective motor control of the lower extremity. It is a clinically applicable tool and does not require any special equipment. It covers the selective movements of the hip, knee, ankle, subtalar, and toe joints bilaterally and individually for each joint. The SMC of each joint was scored as “normal” (2 points) if the patient was able to move the tested joint in isolation (e.g., without moving other joints), within at least 50% of the possible range of movement and at a physiological cadence indicated verbally by the assessor (e.g., “flex, extend, flex”). If any change in performance occurred (slowing of movement, less than 50% of the range of movement, with mirrored/synergic movements), selectivity was considered “impaired” (1 point). An “unable” score was given if no joint movements could be made or if mass synergy patterns occurred.

In addition, a full physical examination was carried out, focusing on the manifestations of spasticity and contractures, which were filled in on the descriptors. Some qualitative factors found in cases with impaired SMC are included in the descriptors. These include the presence of mirrored movements, synergistic movement of joints, slowing down and decreased range of movement.

### 2.2. Translation and Cross-Cultural Adaptation

The translation and cross-cultural adaptation followed the internationally recommended linguistic validation process [14,15,16]. Formal authorization from the authors of the original version was obtained via email before starting the study.

First, the instrument was translated from English into Portuguese by two independent translators that were fluent native speakers of Portuguese. These translators compared their versions and created a unique one. Then, the synthesized questionnaire was backtranslated into the original language by an independent translator who was a fluent native speaker of English (blinded to the original questionnaire). After that, the backtranslated version was sent to the original authors for verification and approved by email.

Once approved, validation procedures were applied for the selected patients. The selection process was performed on all patients with spastic cerebral palsy in our outpatient group; however, only patients who adhered to the inclusion criteria were included.

### 2.3. Participants

Patients from the children’s orthopedics and reconstruction clinic of a public Hospital in São Paulo with a diagnosis of spastic CP, older or equal than four years, with GMFCS levels ranging from I to IV and ability to follow simple commands were recruited. If orthopedic intervention was performed in the last six months or the patient had an inability to consent, the patient was excluded.

The sample size calculation was performed using the OpenEpi platform (version 3), specifically the open-source SSPropor calculator (http://openepi.com/SampleSize/SSPropor.htm; (accessed on 20 February 2025)). Using the population prevalence of cerebral palsy (2:1000), the target population (N = 2,532,053; IBGE 2023), a 95% confidence level (Z = 1.96), absolute precision (d = [specify]), and a design effect (DEFF = 2) appropriate for a non-probability sample, the calculated minimum sample size was 30 participants.

### 2.4. Evaluator Training and Standardization

The evaluators were the three pediatric orthopedics residents at HSPE, who had studied the SMC and trained in the application of SCALE. In addition to the material presented in the original article, we used the instructional video from the Center for Cerebral Palsy Studies at the University of California, Los Angeles (UCLA), to demonstrate the tool’s practical application in English; the video is freely available on Vimeo (https://vimeo.com/438735219; (accessed on 20 February 2025)). The theoretical component and hands-on practice were conducted individually by an experienced pediatric orthopedic surgeon during outpatient clinics for patients with cerebral palsy over the course of one week, approximately 2 h of training.

### 2.5. Assessment

The approach to SCALE-BR test is meticulously detailed and focused on patient comprehension and performance. Initially, the examiner assesses the patient’s ability to follow simple commands and evaluates the passive range of motion (ROM) of each joint prior to active testing. To ensure clarity, each movement is demonstrated while supporting the limb, accompanied by general instructions that emphasize movement isolation and the importance of communicating any questions or concerns. Verbal commands using colloquial terms to facilitate comprehension were used. The examples included in the instructions:

Hip—Ask the patient to flex, extend then flex the hip while keeping the knee extended. For example: “Move your leg forward, back then forward again while keeping your knee straight. I will take you through the motion first, and then I’d like you to do it yourself.”

Knee—Ask the patient to extend, flex then extend the knee while keeping the hip flexed. For example: “Straighten your knee as much as you can, then bend it and straighten again. Try to do this without leaning further back or moving your other leg. I will take you through the motion first, and then I’d like you to do it yourself.”

Ankle—Ask patient to dorsiflex, plantar flex then dorsiflex the ankle while maintaining knee extension. For example: “Keeping your knee straight while I support your leg, move your foot up, down then up again. I will take you through the motion first, then I’d like you to do it yourself.”

Toes—Ask patient to flex, extend then flex toes without moving ankle or knee. For example: “Curl all your toes down, then up then down again while I support your leg. I will take you through the motion first, then I’d like you to do it yourself.”

During execution, the examiner guides the movement speed using a three-second verbal count, allows for multiple attempts, and provides feedback to optimize patient performance, thereby seeking maximal cooperation. Subsequently, the examiner positions the patient specifically for each joint to be tested—the hip (lying on their side), and the knee, ankle, foot/subtalar, and toes (seated at the edge of the examination table)—adapting support as necessary to compensate for muscle tension. Synergy tests may also be applied to investigate specific weaknesses. Upon completion of each joint test, the examiner assigns a score from 0 to 2 points, based on the ability to isolate movement, the achieved range, the speed of execution, and the absence of compensatory or mirror movements, recording all applicable descriptors to comprise the total limb score.

Descriptors evaluation and Physical examination: Evaluators documented qualitative descriptors on standardized forms by marking their presence or absence, specifying the joint topography involved, and noting key details of the movement performed; items captured included mirrored movements, synergistic coupling across joints, and slowing or reduced amplitude of motion, with some descriptors treated as binary (e.g., contractures, localized muscle tension, focal spasticity, mirror movements, slow velocity, low amplitude, or movements conjugated with other joints), while direction-specific features were explicitly described (e.g., inversion, eversion, dorsiflexion, flexion, extension). These descriptors were not graded on a separate standardized ordinal scale; rather, they functioned as a protocolized extension of the physical examination tailored for patients with cerebral palsy. In the final interpretation, descriptors were used as modifying factors to help distinguish patients with similar total SCALE scores and comparable GMFCS levels. However, their weighting and integration have not yet been standardized for unified scoring and warrant future study.

The comprehensive musculoskeletal exam included targeted contracture testing of the hip (rectus femoris, psoas, adductors), knee (medial and lateral hamstrings), and ankle (triceps surae), performed with standard maneuvers. The presence or absence of contracture was recorded in the form and medical chart, and range-of-motion degrees were documented using the instrument’s fields. Standardized spasticity scales, such as the Modified Ashworth Scale, were not applied.

### 2.6. Validity and Reliability

Validation was achieved by looking for a correlation between the new variable under study and the variable considered to be the gold standard. In this case, the expanded and revised edition of the Gross Motor Function Classification System (GMFCS E&R) was used as a comparative factor. The GMFM consists of 88 items categorized into five gross motor function dimensions: Lying and Rolling (A); Sitting (B); Crawling and kneeling (C); Standing (D); and Walking, running, and jumping (E).

In addition, the results of this study were compared with those obtained in the original article by Fowler et al. [6] and four other translations that have already been validated [6,17,18,19,20].

Although the SCALE and GMFCS measure different aspects of the patient’s motor disability, it is expected that individuals with higher SCALE scores will have less impairment of lower limb function, resulting in a higher level of mobility and a lower GMFCS level.

The reliability of the instrument was assessed using the intraclass coefficient, which evaluates the intra- and interobserver ratio. The former represents the reliability of the application of the instrument at different times by the same evaluator, while the latter assesses the reliability between different evaluators in relation to the same patient.

Internal consistency was also assessed, which represents the extent to which a survey, questionnaire, or test can measure. The higher the internal consistency, the more reliable the test result. The α-Cronbach measure was used for this assessment.

### 2.7. Statistical Analysis

Descriptive statistics (mean, standard deviation (SD), range) summarized demographic, GMFCS Scores and SCALE results. SCALE was validated by comparing it with the GMFCS using Spearman’s coefficient. Reliability was assessed using the intraclass correlation coefficient (ICC) model, a two-way mixed-effects model for single measures. Data were centered and scaled using a pooled mean and SD. ICC values were interpreted as follows: <0.40 (poor), 0.40–0.59 (fair), 0.60–0.74 (good), and ≥0.75 (excellent) [21]. The internal consistency results were interpreted as standardized by original article [22]: Group differences in SCALE and GMFCS scores were analyzed via Kruskal-Wallis test (non-parametric test) and were used to evaluate the necessity of a post-hoc analysis, and to this, a Dunn’s post-hoc test was selected to control for the inflated Type I error rate that occurs when performing multiple comparisons, and the Bonferroni correction was applied to the *p*-values for adjustment. All analyses were conducted using R^®^ (version 4.4.0., R Foundation for Statistical Computing), with two-tailed *p*-values and significance set at *p* < 0.05.

## 3. Results

The demographic data are summarized in Table 1. Thirty patients with a diagnosis of spastic cerebral palsy were analyzed, with a predominance of males (66.7%), a mean age of 12.9 ± 7.9 (4–38 years), and most patients were classified as diparetic (73.3%) and GMFCS I (53.3%).

Although the original study included more than 50 participants, the sample size for the present study was clearly smaller. As the primary objective was to validate and standardize the method, we deemed the sample adequate to estimate the measures of interest with the planned precision. Nonetheless, we recognize that the smaller sample size relative to the original study reduces power to detect small effect sizes, limits subgroup analyses, and restricts the generalizability of the findings.

### 3.1. Validity

**Figure 1:**
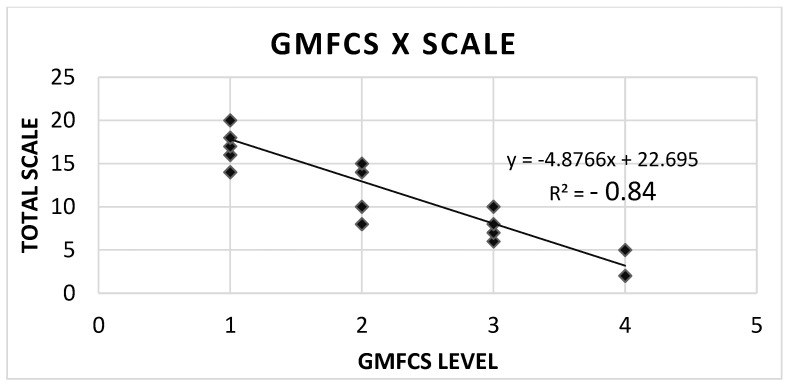
Correlation between GMFCS level and SCALE scores.

The SCALE scores and GMFCS levels showed a high inversely proportional correlation; in other words, the worse the SCALE score, the higher the GMFCS. This was shown by the strong negative result of Spearman’s correlation coefficient, R^2^ = −0.84, *p* < 0.001 (Figure 1).

After the Kruskal-Wallis nonparametric test revealed a statistically significant difference between the groups (*p*-value < 0.0001), it became essential to identify which specific pairs of groups differed from each other. For this purpose, Dunn’s post-hoc test was used. The results of this adjusted Dunn test indicated that Group 1 differs significantly from Group 3 (*p* = 0.003) and from Group 4 (*p* = 0.0015), while the other comparisons between groups did not show notable statistical differences (*p* > 0.05).

Considering the strong inverse correlation between SCALE and GMFCS, where higher SCALE scores correspond to lower GMFCS (less impairment), the identification of significant differences between Group 1 vs. Group 3 and Group 1 vs. Group 4 (with *p* < 0.05) directly reinforces the discriminatory validity of SCALE-BR. This means that SCALE-BR can clearly differentiate patients with mild motor impairment (probably GMFCS I, corresponding to Group 1) from those with moderate (GMFCS III, corresponding to Group 3) and severe (GMFCS IV, corresponding to Group 4) impairments. In short, the post-hoc analysis not only confirms the existence of differences, but also specifies them, strengthening confidence in the SCALE-BR’s ability to distinguish between different levels of gross motor function.

The data in Table 2 demonstrates a clear inverse relationship between selective motor control (SCALE) and gross motor functional severity (GMFCS). As functional impairment increases across GMFCS levels I to IV, mean SCALE scores decrease markedly: 17.6 ± 3.4 (range 9–20) in GMFCS I, 12.4 ± 3.2 (8–15) in GMFCS II, 7.7 ± 1.6 (6–10) in GMFCS III, and 4.0 ± 1.7 (2–5) in GMFCS IV. The ranges show strong discriminative separation: GMFCS IV (2–5) does not overlap with III (6–10), and III only minimally overlaps with II (8–15), while II partially overlaps with I (9–20). This monotonic decline and limited inter-level overlap support the construct validity of SCALE as a measure of selective motor control that worsens with increasing gross motor impairment. Clinically, higher GMFCS levels correspond to more pervasive deficits in isolated joint control captured by SCALE, indicating that SCALE can help stratify severity, inform goal setting, and monitor change over time.

The standard deviations for SCALE scores are smaller at GMFCS levels III (1.6) and IV (1.7) compared to levels I (3.4) and II (3.2), indicating a tighter concentration of scores among patients with more severe motor impairment. While the authors acknowledge some overlaps in SCALE score ranges between adjacent GMFCS levels (partial between I and II, minimal between II and III, and none between III and IV), this overlap does not diminish SCALE’s utility. Instead, it suggests SCALE provides valuable discrimination by offering finer details about selective motor control within each broad GMFCS classification, thus complementing GMFCS by explaining variability and providing additional insights for planning and monitoring that are not captured by GMFCS alone.

### 3.2. Reliability and Internal Consistency of the Questionnaire

Six children were assessed on the application of the Portuguese version of the SCALE on two different occasions. Inter-observer reliability was evaluated between the tests applied by the three evaluators for the total number of patients included. The intra- and interobserver evaluation for the score for each lower limb was good and excellent. The detailed scores are shown in Table 3.

Although the ICCs are high, the confidence intervals in some cases are very wide, with minimum values of 0.15 and 0.24 for interobserver and intraobserver reliability on the right side. The lower sample in this study was adequately powered to detect intraclass correlation coefficients (ICCs) in the good-to-excellent range, despite relatively wide confidence intervals. These findings support the reproducibility of the SCALE BR and suggest that larger samples or multicenter replications could yield narrower confidence intervals.

Regarding internal consistency, a α-Cronbach’s of 0.89 was found for the SCALE on the right and 0.95 on the left, which is an excellent result. Therefore, the items used in the questionnaire have high internal reliability.

## 4. Discussion

The assessment of selectivity before the development of the SCALE questionnaire was used in a non-standardized way [8,9]. Measurement scales such as the “Boyd and Graham test” [23] and the “Trost test” [24] were created and validated but had little reproducibility between observers [25]. Other authors such as Rose et al. [26] used complementary methods for this assessment; in this case, ankle electroneuromyography. However, they had the limitation of not assessing the entire lower limb and, although they had good sensitivity, they did not allow for a grading of the severity of SMC impairment.

SCALE has become a relevant form of clinical assessment with satisfactory intra- and inter-observer results and the ability to measure SMC quantitatively. Furthermore, good SCALE results have been found when related to scores including: Fugl-Meyer Assessment, Manual Muscle Test and Modified Ashworth Scale [27] and Pediatric Balance Scale [28]. importance lies in its value in selecting patient candidates for selective dorsal rhizotomy and predicting functionality, mainly by assessing ankle movements [8,9,12].

The translation process presented no difficulties, and the SCALE-BR questionnaire was considered reliable and valid. High inter- and intraobserver reliability scores support the clinical usefulness of this tool. This instrument has low complexity, clear and concise assessment instructions and classification criteria. However, as for the ability to follow motor commands correctly, patients under the age of four years and those with severe motor or intellectual disorders (GMFCS level V) or other non-spastic forms of cerebral palsy are unable to be approached with this type of assessment.

SCALE-BR showed a significant inverse correlation with GMFCS level (reported as R^2^ = −0.84), echoing the results of the original SCALE. In addition, the average SCALE scores declined from 18.5 among participants with GMFCS level I to 3.5 among those with GMFCS level IV, further illustrating this inverse relationship. These findings align with Fowler et al. [13] and support the view that SMC is an important indicator of disease severity. Consistently, Ostenjø et al. [9] identified SMC as a key factor underlying functional mobility limitations in spastic CP. The quantitative association between SCALE and gross motor function (GMFM-66) further indicates that SMC exerts a stronger influence on gross motor performance in CP than muscle volume or spasticity [29].

When compared with the other studies [17,18,19,20], the main differences lie in the research focus (validation versus exploration of correlations), the demographic and severity characteristics of the samples, the additional validation methodologies employed, and nuances in the findings. Regarding the sample, factors such as the age range (4–38 years in the SCALE-BR versus predominantly children and adolescents in the others, except the Japanese study) and the distribution of GMFCS levels may explain variations in the absolute values of mean SCALE scores across GMFCS groups, as observed in the Japanese study. The Korean study [20] stands out for its aim of exploring the relationship between trunk control and SCALE, whereas the Chinese study [19] goes deeper into the facto structure. The Turkish [18], Japanese [17], and SCALE-BR studies focus more directly on comprehensive psychometric validation of the scale in their respective languages, each contributing data from a unique population and expanding the evidence that SCALE is a globally applicable tool.

Across multiple languages and cultural contexts—including Turkish, Chinese, Korean, and Japanese versions, and now the Brazilian version (SCALE-BR)—SCALE has consistently demonstrated excellent psychometric properties [17,18,19,20]. All studies have confirmed high intra- and interrater reliability, as well as strong internal consistency, ensuring that SCALE is a stable and reproducible tool regardless of the evaluator. More importantly, an inverse and significant correlation between SCALE scores and Gross Motor Function Classification System (GMFCS) levels have been universally observed (r ranging from −0.76 to −0.87), evidencing the scale’s convergent validity in assessing the severity of cerebral palsy. Although small variations may exist in the absolute values of mean SCALE scores across GMFCS levels within each population studied, the instrument’s discriminative trend in differentiating levels of motor impairment—and, in some cases, CP subtypes—has remained unchanged, solidifying SCALE as a cross-culturally valid and clinically relevant instrument for evaluating selective motor control of the lower extremities.

Evaluating the descriptors included in the SCALE makes it possible to ascertain the presence of secondary alterations, such as hip or knee contractures and equinus deformity. In this way, it can be a guide to direct the treatment of these findings, for example, botulinum toxin for spasticity or surgical interventions for lower limb contractures. Studies are still needed to assess the correlation of the SCALE with the progression and severity of the descriptors.

It is important to highlight the role that the SMC plays in human gait, being responsible for the uncoupled or nonsynergistic movements of the limbs. This activity allows for a refined stride, i.e., knee extension associated with hip flexion and ankle dorsiflexion to advance the step, which occurs during the swing phase and would not occur without the SMC [30]. Further evidence from gait analysis studies, such as those by Chruscikowski et al. [31], Zhou et al. [32], and Sardoğan et al. [33], consistently demonstrates a negative correlation between SCALE scores and overall gait impairment. Individuals with greater SMC impairment, as measured by SCALE, exhibit less complex neuromuscular control and increased flexor/extensor synergy during gait [30]. Importantly, impaired SMC predicts increased knee flexion at initial contact, decreased step length, and reduced gait speed in children with spastic CP [31], and is visibly related to foot and ankle movements during the swing phase and initial posture during walking [33].

Understanding the role of SMC is paramount for improving the prognosis and tailoring interventions for patients with CP, especially those with similar GMFCS who exhibit varied outcomes. SMC acts as a critical modifying factor influencing the success of various procedures and therapies. While comprehending its impact on the clinical profile and gait mechanism can significantly guide intervention indications, from intensive physiotherapy programs to surgical procedures [5,10,31,32,33], it is crucial to recognize the current limitations of the SCALE-BR. Specifically, it does not presently offer explicit score thresholds that dictate treatment choices, such as when to opt for botulinum toxin over intensive physiotherapy, or when surgical intervention becomes the more suitable option, serves as a complementary assessment tool within a broader clinical evaluation. This foundational importance of SMC is particularly evident in procedures like Selective Dorsal Rhizotomy (SDR): although SDR aims to reduce spasticity, significant functional recovery after the procedure depends on the patient’s ability to take advantage of the reduction in spasticity to perform voluntary and coordinated movements [7,11]. SMC is the basis for functional movement; if a patient does not have that, even with decreased spasticity, the quality and usefulness of movements may be limited. Consequently, while SMC is a key prognostic indicator, there is currently no formal mechanism within SCALE-BR to directly monitor prognosis. This underscores the imperative for future responsiveness studies to ascertain whether SCALE-BR scores accurately reflect appropriate changes following specific interventions.

This instrument, while valuable, is subject to several important limitations. Its validation study was based on a small sample size of only 30 patients, which significantly limits the statistical power for detailed subgroup analyses and thus impacts the overall generalizability of the findings. Moreover, the sample predominantly featured individuals with milder forms of cerebral palsy, thereby restricting the tool’s proven applicability to more severely affected populations. The cross-sectional design of the study further confines the conclusions that can be drawn, as it prevents assessing the tool’s sensitivity to change or its responsiveness to therapeutic interventions over time. Additionally, recruitment from a single site within Brazil means the results may lack broad geographic generalizability.

## 5. Conclusions

The SCALE-BR questionnaire was effective, proving to be reproducible and reliable between different evaluators and patients, with an inverse correlation with the GMFCS that is consistent with existing literature, underscoring its convergent validity and clinical utility as a measure of disease severity. This robust initial validation positions SCALE-BR as a valuable and accessible tool for clinicians and researchers. To further enhance the SCALE-BR’s utility, future research should focus on several key areas: conducting responsiveness studies to confirm its ability to track changes after interventions, performing three-dimensional gait analysis correlation studies to understand biomechanical implications of SMC impairments, investigating its predictive validity for long-term functional outcomes, and carrying out cultural validation across diverse Brazilian socioeconomic and educational backgrounds to ensure equitable application. These efforts will deepen the understanding of SMC in cerebral palsy and empower clinicians with a more comprehensive tool for assessment, intervention, and prognosis.

## Figures and Tables

**Table 1:** Demographic and pathophysiological data.

Variable		Mean ± SD (Range)
Age		12.9 ± 7.9 (4–38 years)
Gender		
	Male	20 (66.7%)
	Female	10 (33.3%)
Level Distribution		
	Diparetic	22 (73.3%)
	Hemiparetic	5 (16.7%)
	Quadriparetic	3 (10.0%)
GMFCS		
	I	16 (53.3%)
	II	5 (16.7%)
	III	6 (20.0%)
	IV	3 (10.0%)

**Table 2:** SCALE scores based on GMFCS.

	GMFCS I	GMFCS II	GMFCS III	GMFCS IV
SCALE (mean ± SD (range))	17.6 ± 3.4 (9–20)	12.4 ± 3.2 (8–15)	7.7 ± 1.6 (6–10)	4.0 ± 1.7 (2–5)

**Table 3:** Intra- and inter-observer reliability (ICC) of the SCALE.

	Side	Reliability	95% CI
ICC Inter-observer (1.1)	Right	0.82 *	0.24–0.96
	Left	0.92 *	0.56–0.98
ICC Intra-observer (2.1)	Right	0.78 *	0.15–0.96
	Left	0.90 *	0.72–0.97

* *p*-value < 0.001.

## Data Availability

The data presented in this study are available on request from the corresponding author due to privacy and ethical reasons.

## Abbreviations

The following abbreviations are used in this manuscript:

IAMSPE	Instituto de Assistência Médica ao Servidor Público Estadual
HSPE	Hospital do Servidor Público Estadual
SCALE	Selective Control Assessment Lower Extremity
CMS	Controle Motor Seletivo
PC	Paralisia Cerebral
